# Isolation of antigen-specific CD8+ T lymphocytes in vitro and in vivo

**DOI:** 10.1186/2051-1426-1-S1-P239

**Published:** 2013-11-07

**Authors:** Carina Wehner, Christian Ellinger, Silke Raffegerst, Susanne Wilde, Barbara Mosetter, Dolores J Schendel, Slavoljub Milosevic

**Affiliations:** 1 Institute of Molecular Immunology, Helmholtz Center Munich, Munich, Germany

## 

Depending on the mechanisms by which cancer develops and the strategies tumors use to escape the immune system, cancer therapies are more or less successful. A combination of different therapeutic agents, such as dendritic cell (DC) vaccines loaded with tumor antigens and adoptive transfer of tumor-specific T-cells, may enhance the chances of effective cures. Thus, for the isolation of T-cell receptors (TCR) that can be transferred into patient-derived lymphocytes, enabling T-cells to recognize and eliminate tumor cells, we induced tumor-associated-antigen (TAA)-specific T-cells by loading DC with in vitro transcribed (ivt) RNA coding for 5 different cancer-testis-antigens (CTA, GAGE-1, MAGE-A4, NY-ESO-1, SSX4 and XAGE-1). As CTA are self-antigens with a limited expression in immune privileged tissues and in various tumor entities, we needed to bypass negative selection of highly avid T-cells specific for CTA restricted by self-MHC molecules. Therefore, allo-reactive T-cells, which are able to recognize antigens on foreign MHC molecules, offer the possibility to gain high-affinity TCR. Using DC and responding PBL from two donors that expressed different MHC allotypes resulted in the presentation of CTA on several different MHC molecules encoded by endogenous HLA genes. In addition, taking into account that CD4+ T cell help is considered to be essential for the activation and expansion of antigen-specific CD8+ T-cells, target antigens were fused to cell internal localization signals (targeting) which led to presentation by both MHC-I and -II molecules of antigen-presenting cells, allowing the simultaneous activation of CD4+ and CD8+ T-cells. By this means, several CTA-specific T-cell clones were identified. Furthermore, we were able to demonstrate a clear benefit of using DC transfected with targeting-linked Melan-A-ivt-mRNA compared with DC transfected with standard Melan-A-ivt-mRNA in a humanized in vivo DC vaccination model. NOD/scid IL2Rγnull (NSG) mice are deficient for T-, B- and NK-cells, providing a niche for engraftment of human peripheral blood mononuclear cells (PBMC). Vaccination of reconstituted mice with DC loaded either with targeting-Melan-A-ivt-mRNA or with Melan-A-ivt-mRNA resulted in a superior induction-capacity of targeting-Melan-A-ivt-mRNA-transfected DC, demonstrated by significantly greater activation and proliferation of Melan-A-specific CD8+ T-cells. In conclusion, we were able to induce tumor-antigen specific CD8+ T-cells in vitro and in vivo by using targeting-linked antigens provided to DC. Since adoptive T-cell transfer and DC vaccination hitherto have failed to be optimally efficacious in clinical trials, the combination of these two approaches may provide greater clinical benefit to more patients.

